# Habitat partitioning, co-occurrence patterns, and mixed-species group formation in sympatric delphinids

**DOI:** 10.1038/s41598-023-30694-w

**Published:** 2023-03-03

**Authors:** Jonathan Syme, Jeremy J. Kiszka, Guido J. Parra

**Affiliations:** 1 Cetacean Ecology, Behaviour and Evolution Lab, College of Science and Engineering, Flinders University, Adelaide, SA Australia; 2 Institute of Environment, Department of Biological Sciences, Florida International University, North Miami, FL USA

**Keywords:** Behavioural ecology, Community ecology

## Abstract

Numerous species have been reported to form mixed-species groups, however, little is known about the interplay between niche partitioning and mixed-species group formation. Furthermore, it is often unclear whether species come together by chance due to overlapping habitat preferences, by shared attraction to resources, or by attraction between them. We assessed habitat partitioning, co-occurrence patterns, and mixed-species group formation of sympatric Australian humpback (*Sousa sahulensis*) and Indo-Pacific bottlenose dolphins (*Tursiops aduncus*) around the North West Cape, Western Australia, with a joint species distribution model and temporal analyses of sighting data. Australian humpback dolphins preferred shallower and more nearshore waters than Indo-Pacific bottlenose dolphins, yet these species co-occurred more often than expected by chance given shared responses to environmental variables. Indo-Pacific bottlenose dolphins were sighted more often than Australian humpback dolphins during the afternoon, however, we did not find any temporal patterns in the occurrence of mixed-species groups. We propose that the positive association in the species’ occurrence indicates the active formation of mixed-species groups. By evaluating habitat partitioning and co-occurrence patterns, this study provides direction for future work which should proceed to investigate the benefits that these species may gain from grouping with each other.

## Introduction

Ecologically similar species that co-occur and share similar habitats typically display some degree of niche partitioning as natural selection favours traits that reduce competition^1–5^. By consuming different resources or by utilising resources in different places or at different times, sympatric species manage to coexist^2,3^. The degree of niche partitioning between species affects species diversity and community composition and, therefore, is a critical aspect of community ecology^1,2^. Where species’ niches overlap, they may occur within close spatiotemporal proximity and even form mixed-species groups.

Mixed-species groups have been reported amongst diverse species, from rainforest birds and primates to oceanic cetaceans^6–9^. They occur when individuals of multiple species actively achieve and maintain spatiotemporal proximity due to a mutual or unilateral attraction. This attraction stems from the evolutionary benefits that can be gained from grouping with heterospecifics (e.g., reduced predation risk, enhanced foraging, and the opportunity to interact socially with heterospecifics)^6,7,9,10^. The stability, frequency, and benefits of mixed-species groups can be influenced by the species’ habitat use and patterns of co-occurrence^8,9,11–13^. For example, as a result of utilising different forest strata when in mixed-species groups, primates may exhibit complementary predator vigilance (e.g., those in lower strata are more vigilant to terrestrial threats while those in higher strata are more vigilant to arboreal and aerial threats) resulting in decreased predation risk for the group^8,11^, while differences in dietary niche mean that birds that form mixed-species groups can gain the antipredator benefits of grouping while experiencing less severe competition for food than they would in single-species groups^12,13^.

Within a given ecosystem, species from the same trophic guild can co-occur at high densities and share similar habitats. Therefore, individuals of different species may periodically encounter each other by chance^8,9,14,15^. Such chance encounters, however, are unlikely to have any evolutionary significance^15,16^. Where species utilise the same localised resources (e.g., food pulses or resting areas), encounters between individuals of multiple species may occur more frequently. In this case, their co-occurrence is best described as an aggregation as it results from a shared, but independent, attraction to a given resource^6,9,17^.

Although they may appear outwardly similar, chance encounters and aggregations should be clearly distinguished from mixed-species groups based on functional benefits^6,9,10^. More specifically, this involves determining whether there is attraction amongst individuals (i.e., they truly form a group) or if their occurrence in spatiotemporal proximity is simply a chance encounter due to overlapping habitat preferences or an aggregation due to shared attraction to resources^6,14,15^. During chance encounters and aggregations, individuals of multiple species occur in close proximity and may exhibit similar behaviours. As a result, they may appear to form a cohesive group and they may even meet the various criteria that are used by researchers to define groups in the field (e.g., spatial proximity and behaviour)^10,18,19^. Consequently, various analytical methods have been proposed to confirm that species observed together in apparent mixed-species groups are indeed brought together by attraction.

The ideal gas model, for example, has been used to study mixed-species groups of primates^14,16,20^. This method estimates expected encounter rates by simulating the movements of groups through space. However, it requires detailed information on group travel speed, diameter, and density that are not always readily obtainable, particularly for wide-ranging marine species^8^. Alternatively, presence-absence data of sympatric species can be analysed with null model randomisation tests to determine if species co-occurrence rates are significantly above or below what would be expected by chance^21^. Although null models have been used to analyse the co-occurrence rates of sympatric species^22,23^, they do not consider the possibility that non-random patterns of co-occurrence result from shared responses to environmental features^24,25^.

More recently, joint species distribution models (JSDMs) have been developed to address this issue^26^. By simultaneously modelling multiple species’ responses to both environmental factors and to heterospecific presence, JSDMs can separate correlations in species occurrence into that which is due to environmental factors (i.e., environmental correlation) and that which is unexplained (i.e., residual correlation)^25,27,28^. The residual correlation can be the result of either non-measured environmental or biotic factors or interactions between the species (e.g., avoidance or attraction). However, as environmental factors and species interactions can, in theory, generate identical presence-absence data, these two possibilities are statistically indistinguishable^24,29^. Despite this limitation, JSDMs can effectively identify non-random relationships between species while accounting for the influence of measured environmental factors^27,30^. Thus, JSDMs can determine whether species are found together in close spatiotemporal proximity by chance, provide inferences about potential biotic interactions, including attraction, and provide evidence for niche partitioning^27,28^.

JSDMs have been used, for example, to determine the influence of biotic interactions and environmental responses on the habitat partitioning of mountain birds^31^ and the composition of stream macroinvertebrate communities^32^. Their use in the study of delphinids is currently limited, although they have been used to evaluate the factors that drive the distribution of marine predators, including common (*Delphinus delphis*) and striped dolphins (*Stenella coeruleoalba*), in the Bay of Biscay^33^. The ability of JSDMs to identify non-random relationships between species after accounting for environmental responses makes them a potentially useful tool in the study of mixed-species groups. For example, they have been used to detect positive associations between fish species in mixed-species groups and, subsequently, to assess the impact of heterospecific social information on assemblage patterns^30^. Nevertheless, the application of JSDMs to the study of mixed-species groups remains underutilised.

Many delphinid species co-occur in the same habitat and even form mixed-species groups, however the mechanisms promoting their coexistence and the drivers of mixed-species group formation are poorly understood^6–8,34–37^. Previous studies have hypothesised that delphinids form mixed-species groups for a variety of reasons, which, broadly speaking, correspond to three proposed functional explanations: to improve foraging, to reduce predation risk, and to gain social benefits^6–8^. Most studies of delphinid mixed-species groups make the assumption, based on observed behaviours or the presumed improbability of chance encounters, that apparent mixed-species groups are the result of an attraction between species^6–8^. Accordingly, the possibility that delphinids could come together, not by attraction between species, but rather by chance due to shared use of space or by shared attraction to resources has rarely been explicitly tested^6–8^. Moreover, previously used approaches are limited to analysis of interspecific social networks to determine non-random patterns of association which requires individual photo-identification^38,39^ and the use of a minimum time limit which relies on an arbitrarily chosen limit to designate encounters between species as random or non-random^40^.

Australian humpback (*Sousa sahulensis*, hereafter “humpback dolphins”) and Indo-Pacific bottlenose dolphins (*Tursiops aduncus*, hereafter “bottlenose dolphins”) overlap in range across the coastal waters of northern Australia^41–43^ with humpback dolphins tending to occupy shallower and more nearshore waters than bottlenose dolphins^44–47^. Around the North West Cape, Western Australia, these two species occur in sympatry and behave in such a way that they meet operational definitions of group that have been applied in the field^41,48^ and, accordingly, appear to form mixed-species groups. However, it has not yet been confirmed that these apparent mixed-species groups are indeed groups that result from attraction between the species and not simply chance encounters or aggregations around common resources^6^.

Here, we investigated spatial and temporal occurrence patterns of humpback and bottlenose dolphins around the North West Cape to assess habitat partitioning and determine whether the species occur together more or less often than expected by chance given their responses to environmental factors. We evaluated the extent and nature of habitat partitioning and co-occurrence between the species both spatially, with a JSDM, and temporally, by analysing their occurrence to detect any diel, seasonal, or yearly patterns in their co-occurrence. We hypothesised that they would display spatial habitat partitioning, with humpback dolphins in shallower water nearer to the coast, but not temporal partitioning. Furthermore, we aimed to quantitatively determine whether there is attraction between these species and, in doing so, test the assumption that humpback and bottlenose dolphins form mixed-species groups. More specifically, we used the JSDM to determine whether the co-occurrence of humpback and bottlenose dolphins is higher than that expected by chance after accounting for shared responses to environmental factors. This study represents an important step in understanding habitat partitioning and coexistence in sympatric species as well as the drivers of the formation of mixed-species groups.

## Methods

### Study site

The North West Cape, Western Australia, is bordered to the west and north by Ningaloo Reef, whose sandy coral lagoons are protected by a shallow reef crest that falls away steeply towards open water, and to the east by Exmouth Gulf, whose shallow, turbid waters contain scattered coral reefs, seagrass meadows, and mangroves^49,50^. The waters of the North West Cape provide important habitat for a diverse array of species, including both humpback and bottlenose dolphins^45,51^.

### Data collection

**Figure 1:**
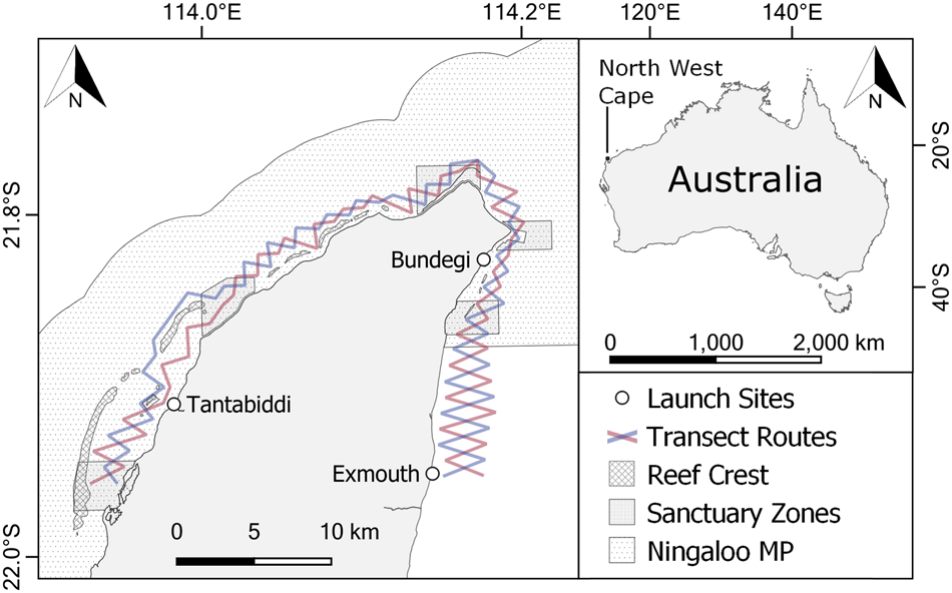
The North West Cape, Western Australia, showing vessel launch sites and two opposing, zigzag transect routes (Route A: blue; Route B: red) used to survey for Australian humpback (*Sousa sahulensis*) and Indo-Pacific bottlenose dolphins (*Tursiops aduncus*).

The study area covered approximately 175 km^2^ of shallow (< 40 m deep), inshore (< 5 km from shore) waters from Exmouth, north around the Cape, to the southern end of South Lagoon (Fig. 1). This area was surveyed repeatedly from a 5.6 m research vessel by following two predetermined, opposing, zigzag transect routes at a constant speed averaging 7 knots (Fig. 1). Surveys were conducted across six austral winter (April to October) field seasons (2013–2015, 2018–2019, and 2021) during daylight hours and optimal survey conditions (i.e., Beaufort scale ≤ 3 and no rain or fog)^51^. Sightings consisted of both single individuals and groups, which were operationally defined as two or more individuals within 100 m of one another and engaged in similar behaviour^19^. Upon each dolphin sighting, relevant data were recorded, including the GPS location, the time, the number of individuals, the predominant initial behavioural state (as per^52^), and the species present.

### Ethics approvals and permits

Data collection was conducted under permit from the Western Australian Government Department of Biodiversity, Conservation and Attractions (formerly Department of Parks and Wildlife) (permit numbers: SF009240, SF009768, SF010289, and FO25000012) and the Australian Government Department of Defence (Naval Communication Station Harold E Holt) with ethics approval from the Flinders University Animal Welfare Committee (project numbers: E383 and E462/17).

### Data preparation

The preparation of all input data for the JSDM (i.e., sites, sighting data, survey effort data, and environmental data) was conducted within the PyQGIS API using Python 3.8.0^53^ and QGIS 3.8.3 Zanzibar^54^. The study area was divided into 540 grid squares (i.e., sites) of 500 × 500 m. These sites formed the basis for the layers of the response variables (i.e., presence-absence of each species) as well as the environmental predictor variables (i.e., water depth and distance to shore) and survey effort (Table 1). This grid size resolution is in line with previous studies on the distribution of inshore dolphins^45,47,55^ and is a balance between coarser resolutions (e.g., 1000 m), which lead to decreased model performance, and finer resolutions (e.g., 100 m), which are more heavily affected by background absences^56,57^. Furthermore, this size is sufficiently small to capture the variation in the habitat characteristics of the study site and corresponds to the spatial criterion used in the group definition which, being a chain-rule, allows for the group members to be spread over a larger area than the distance threshold.

**Table 1:** The predictor variables included in the joint species distribution model of Australian humpback (*Sousa sahulensis*) and Indo-Pacific bottlenose dolphins (*Tursiops aduncus*) around the North West Cape, Western Australia, and their data sources. Values for predictor variables were calculated within the PyQGIS API using Python 3.8.0^53^ and QGIS 3.8.3 Zanzibar^54^.

Predictor variable	Units	Data source
Water depth	m	Water depth for each site was calculated with the Ordinary Kriging Tool (SAGA Toolbox^61^) from in situ measurements (n = 5024) taken with the research vessel’s depth sounder
Distance to shore	m	Distance to shore was measured as the Euclidean distance from the centre of each site to the nearest land
Cumulative survey effort	m^2^	Daily survey effort was calculated by adding a 250 m buffer to the recorded GPS track of the research vessel and then calculating the survey effort area within each site. Cumulative survey effort was calculated by summing the daily survey effort for each austral season (i.e., autumn, winter, and spring)

Binary presence-absence data were generated for each species by plotting the dolphin sightings from each survey day and determining if each species was either present (1) or absent (0) in each site. Survey effort was calculated for each survey day by adding a 250 m buffer to the recorded GPS track of the research vessel and then calculating the survey effort area within each site (Table 1). This buffer distance approximates the reliable visual survey coverage for inshore dolphins from the research vessel. Due to low sighting rates, the daily presence-absence and survey effort data were pooled into three austral seasons: autumn (March—May), winter (June—August), and spring (September—November) (Supplementary Figs. S1 and S2). This was necessary to avoid issues with model convergence caused by zero-inflation^27,58^.

Water depth and distance to shore were included as environmental covariates because both influence the distribution of and demarcate niche partitioning between various dolphin species, including humpback and bottlenose dolphins^45,47,55,59,60^. Most notably, recent research has shown that water depth and distance to shore are the two key factors influencing the distribution of the humpback and bottlenose dolphin populations of the North West Cape^45,47^. Other environmental and anthropogenic factors (e.g., habitat type, sea surface temperature, or distance to boat ramps), on the contrary, were found to have little to no effect^45,47^ and, consequently, were not included in our analysis.

Environmental factors were sampled across the same sites (i.e., 500 × 500 m grid squares) that were used for determining species presence-absence. Distance to shore was measured as the Euclidean distance from the centre of each site to the nearest land and water depth for each site was calculated with the Ordinary Kriging Tool (SAGA Toolbox^61^) from in situ measurements ($n=5024$) taken with the research vessel’s depth sounder (Table 1 and Supplementary Fig. S3). Before conducting the analysis, we tested for collinearity between the environmental variables in R version 3.6.1^62^ and RStudio 1.2.5^63^ with Pearson’s correlation coefficient and a threshold of $\left|r\right|<0.7$^58,64^.

### Joint species distribution model

We analysed the co-occurrence of humpback and bottlenose dolphins around the North West Cape with the Hierarchical Modelling of Species Communities (HMSC) framework^27^ implemented in R version 3.6.1^62^ and RStudio 1.2.5^63^ with the package Hmsc 3.0^65^. The HMSC framework employs hierarchical Bayesian JSDMs and Markov chain Monte Carlo (MCMC) sampling to model the occurrence of species while accounting for environmental filtering, resulting in a residual species association matrix (i.e., the **Ω** matrix)^27,65^.

The response variable of our JSDM consisted of the presence-absence data of humpback and bottlenose dolphins in each season across all 540 sites (the **Y** matrix). Accordingly, a probit regression model was employed with the environmental factors (i.e., water depth and distance to shore) and survey effort included as fixed effects (the **X** matrix). In accordance with the hierarchical nature of the sampling regime, random effects were included for both site and season. Moreover, to account for the spatial arrangement of the sites, the site-level random effect was included as a spatially explicit random effect based on geographic location, defined as the coordinates of the centre of each site.

The posterior distribution was sampled using four MCMC chains of 375,000 iterations each. For each chain, the first 125,000 iterations were discarded as the transient while the remaining 250,000 iterations were thinned by 1000 to produce 250 posterior samples—1000 posterior samples in total. Model convergence was evaluated by assessing the effective sample sizes and by examining the potential scale reduction factors of the model parameters^66^. Model fit was assessed with the area under the curve (AUC)^67^ and Tjur’s R^2^ statistics^68^ for both explanatory and predictive power, calculated with two-fold cross-validation of the model^28,65^. The relative influence of the fixed (i.e., environmental variables and survey effort) and random effects (i.e., site and season) was evaluated with variance partitioning^65^ while habitat partitioning between humpback and bottlenose dolphins was assessed by predicting occurrence probabilities for each species across environmental gradients of water depth and distance to shore while normalising the remaining variables to their mean values^28,65^. The residual association between humpback and bottlenose dolphins (i.e., the omega parameter) was used as the basis to investigate the possibility and nature of interactions between these species^27,28,65^. Parameter estimates were deemed significant if the posterior probability was ≥ 0.95^28^.

### Temporal analysis

We analysed temporal partitioning between the species and temporal variation in the observed frequency of mixed-species sightings across three temporal scales: diel, seasonal, and yearly. For the diel analysis, time of day was separated into morning (0600 to 1000 h), midday (1000 to 1400 h), and afternoon (1400 to 1900 h) while for the seasonal and yearly analyses, each surveyed season (i.e.: autumn, winter, and spring) and year (i.e.: 2013, 2014, 2015, 2018, 2019, and 2021) constituted a time period, respectively. To assess temporal partitioning, we used a chi-square test to compare the number of sightings of humpback dolphins (single- and mixed-species) to the number of sightings of bottlenose dolphins (single- and mixed-species) across the time periods for each temporal scale. We also determined whether the proportion of sightings that were mixed varied over time. Specifically, we used a Fisher’s exact test, due to the low sample size, to compare the number of single- and mixed-species sightings across the time periods for each species at each temporal scale. All temporal analyses were conducted in R version 3.6.1^62^ and RStudio 1.2.5^63^ at a significance level of $\alpha =0.05$.

## Results

### Sightings summary

**Figure 2:**
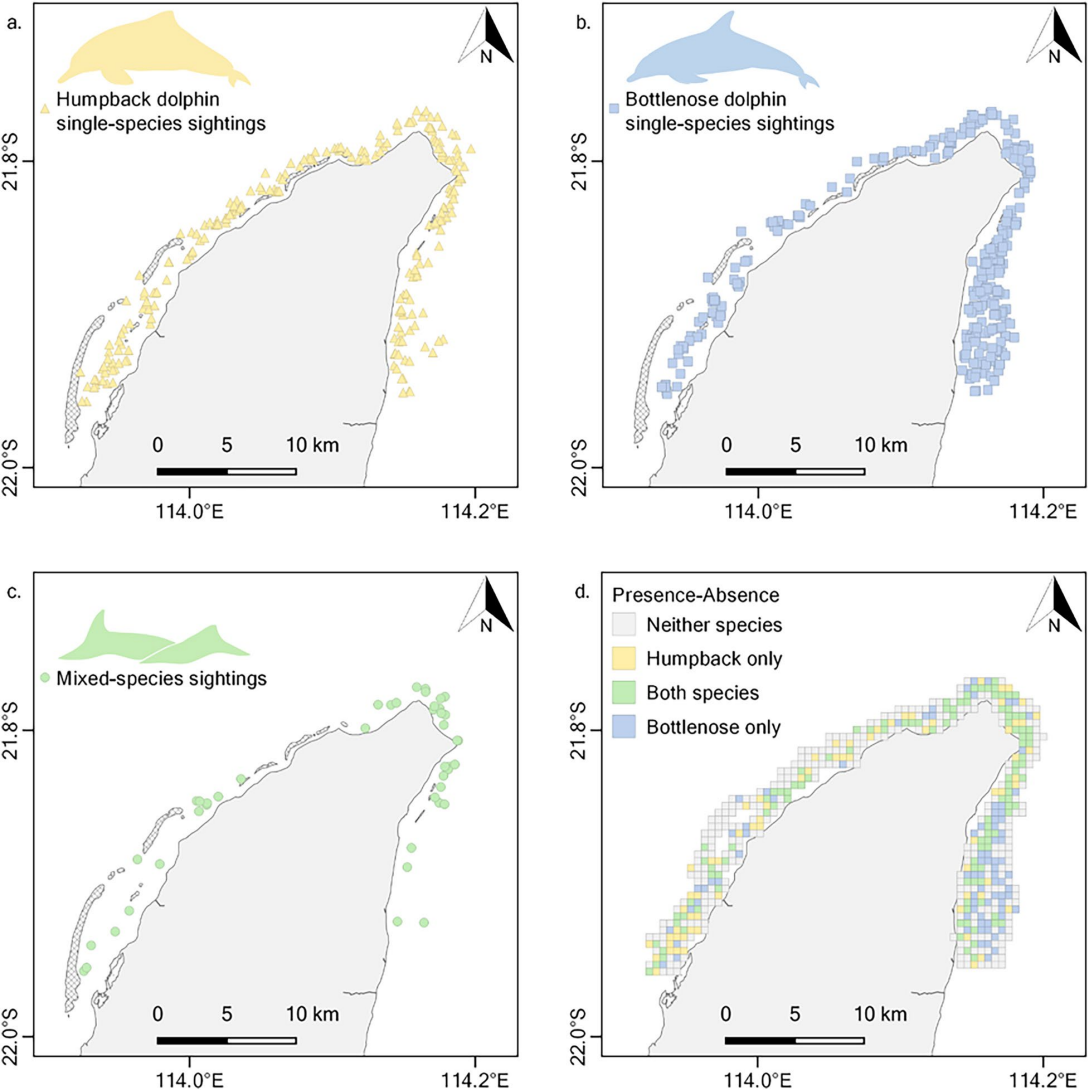
On-effort single-species sightings of (**a**) Australian humpback dolphins (*Sousa sahulensis*) and (**b**) Indo-Pacific bottlenose dolphins (*Tursiops aduncus*), and (**c**) mixed-species sightings of both species, as well as (**d**) overall presence-absence of the species in 540 grids of 500 × 500 m distributed around the North West Cape, Western Australia, from six years of surveys (2013–2015, 2018–2019, and 2021).

In total, 417 days of survey effort were conducted across six years (see Table 2 for a summary of survey effort) resulting in 564 on-effort sightings — 221 of humpback dolphins, 299 of bottlenose dolphins, and 44 of both species (Fig. 2). Thus, mixed-species sightings accounted for 16.6% of all humpback dolphin sightings (single- and mixed-species), 12.8% of all bottlenose dolphin sightings (single- and mixed-species), and 7.8% of all dolphin sightings. The humpback dolphin single-species sightings contained, on average, 4.2 ± 2.4 individuals, bottlenose dolphin single-species sightings contained 5.6 ± 4.6 individuals, and mixed-species sightings contained 8.8 ± 4.9 individuals in total (i.e., both species combined). Of the 44 mixed-species sightings, the most frequent initial behavioural state was travelling (17 sightings, 38.6%), followed by socialising (9 sightings, 20.5%) and resting (9 sightings, 20.5%), then foraging (6 sightings, 13.6%), and milling (2 sightings, 4.5%). Mixed-species sightings were distributed around the study area (Fig. 2) and were recorded in waters from 1.4 to 19.7 m deep, with an average depth of 8.0 m.

**Table 2:** Summary of the effort of surveys for Australian humpback (*Sousa sahulensis*) and Indo-Pacific bottlenose dolphins (*Tursiops aduncus*) around the North West Cape, Western Australia, indicating the number of repeats of the two opposing zigzag transect routes (Route A and Route B: see Fig. 1) and the number of survey days.

Year	Route A	Route B	Survey Days
2013	6	6	73
2014	8	8	83
2015	7	6	68
2018	6	7	69
2019	8	7	85
2021	5	4	39
Total	40	38	417

### Joint species distribution model

Water depth and distance to shore showed a positive collinearity (Pearson’s correlation coefficient, $r=0.56, p<0.001$), but, as it was below the threshold ($\left|r\right|>0.7$)^58,64^, both environmental covariates were included in the JSDM. The HMSC diagnostics indicated good MCMC convergence. For both the beta parameters (i.e., species responses to environmental variables) and the omega parameters (i.e., species associations at the site level), the effective sample sizes were close to 1000, and the potential scale reduction factors were mostly below 1.1 (Supplementary Fig. S4). The model fit was also satisfactory with mean explanatory and predictive power of 0.86 and 0.77, respectively, as measured by AUC, and 0.20 and 0.14, respectively, as measured by Tjur’s R^2^ (Table 3).

**Table 3:** The explanatory and predictive power of the joint species distribution model of Australian humpback (*Sousa sahulensis*) and Indo-Pacific bottlenose dolphins (*Tursiops aduncus*) around the North West Cape, Western Australia, as measured by the area under the curve (AUC) and Tjur’s R^2^ statistics.

Species	Measure	Explanatory	Predictive
Humpback dolphin	AUC	0.83	0.75
	Tjur’s R^2^	0.15	0.10
Bottlenose dolphin	AUC	0.90	0.79
	Tjur’s R^2^	0.27	0.17

**Figure 3:**
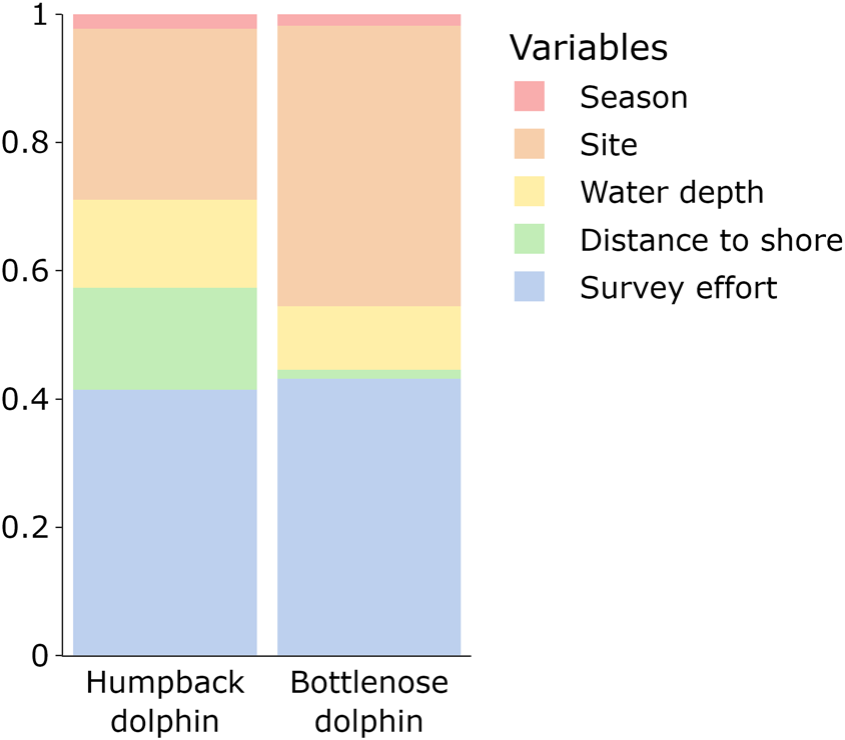
The proportion of variance in the occurrence of Australian humpback (*Sousa sahulensis*) and Indo-Pacific bottlenose dolphins (*Tursiops aduncus*) around the North West Cape, Western Australia, explained by the random effects (i.e., season and site) and the fixed effects (i.e., water depth, distance to shore, and survey effort) included in the joint species distribution model.

**Figure 4:**
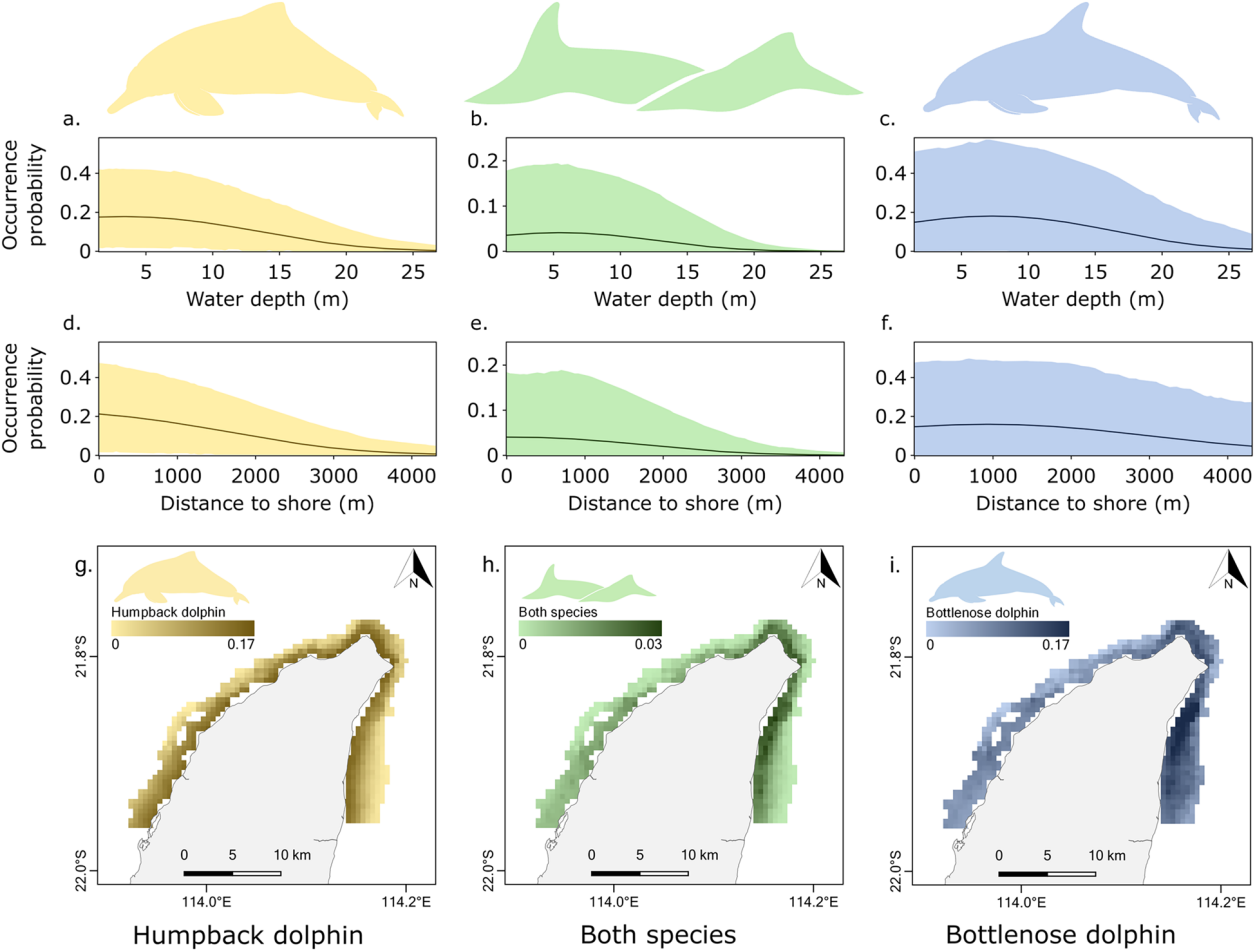
Results of a joint species distribution model of the occurrence of Australian humpback (*Sousa sahulensis*) and Indo-Pacific bottlenose dolphins (*Tursiops aduncus*), around the North West Cape, Western Australia. The three columns illustrate the predicted occurrence probability of humpback dolphins, the predicted co-occurrence probability of the two species, and the predicted occurrence probability of bottlenose dolphins relative to water depth (**a**, **b**, and **c**) and distance to shore (**d**, **e**, and **f**), as well as across 540 grids of 500 × 500 m (**g**, **h**, and **i**). In the first (**a**, **b**, and **c**) and second rows (**d**, **e**, and **f**), the line represents the mean and the shaded area the 95% credibility interval. Occurrence probabilities for humpback and bottlenose dolphins were calculated while normalising the remaining variables to their mean values. Co-occurrence probabilities were calculated by multiplying the corresponding single-species occurrence probabilities.

The different amounts of influence of the fixed and random effects on the occurrence of humpback and bottlenose dolphins were illustrated by variance partitioning (Fig. 3). Survey effort accounted for a substantial amount of explained variance for both species (humpback dolphin, 41.5%; bottlenose dolphin, 43.1%), highlighting that detection rates and, thus, observed occurrence rates are heavily dependent on the amount of survey effort conducted in each site. Distance to shore was highly relevant to humpback dolphins (15.9%), but not bottlenose dolphins (1.4%) whereas water depth was important for both species (humpback dolphin, 13.7%; bottlenose dolphin, 9.9%). The predicted occurrence probability of humpback dolphins was highest at depths of one to five metres and decreased with distance to shore (Fig. 4a,d, and g) while that of bottlenose dolphins was highest at depths of seven to ten metres and peaked at approximately 1000 m from shore before decreasing (Fig. 4c,f, and i). The predicted co-occurrence probability of the species showed intermediate trends, peaking at approximately five metres deep and decreasing with distance to shore (Fig. 4b,e, and h). The spatial random effect (i.e., site) strongly impacted both species (humpback dolphin, 26.7%; bottlenose dolphin, 43.8%), indicating that they display strong preferences for certain sites, while the temporal random effect (i.e., season) had only a minor impact (humpback dolphin, 2.2%; bottlenose dolphin, 1.8%), suggesting that their occurrence is not affected by seasonal changes. Humpback and bottlenose dolphins displayed a strong, positive association in their occurrence with a residual correlation of 0.8 (posterior probability > 95%), indicating that, after accounting for their shared responses to environmental factors, they co-occur more often than expected by chance.

### Temporal analysis

**Figure 5:**
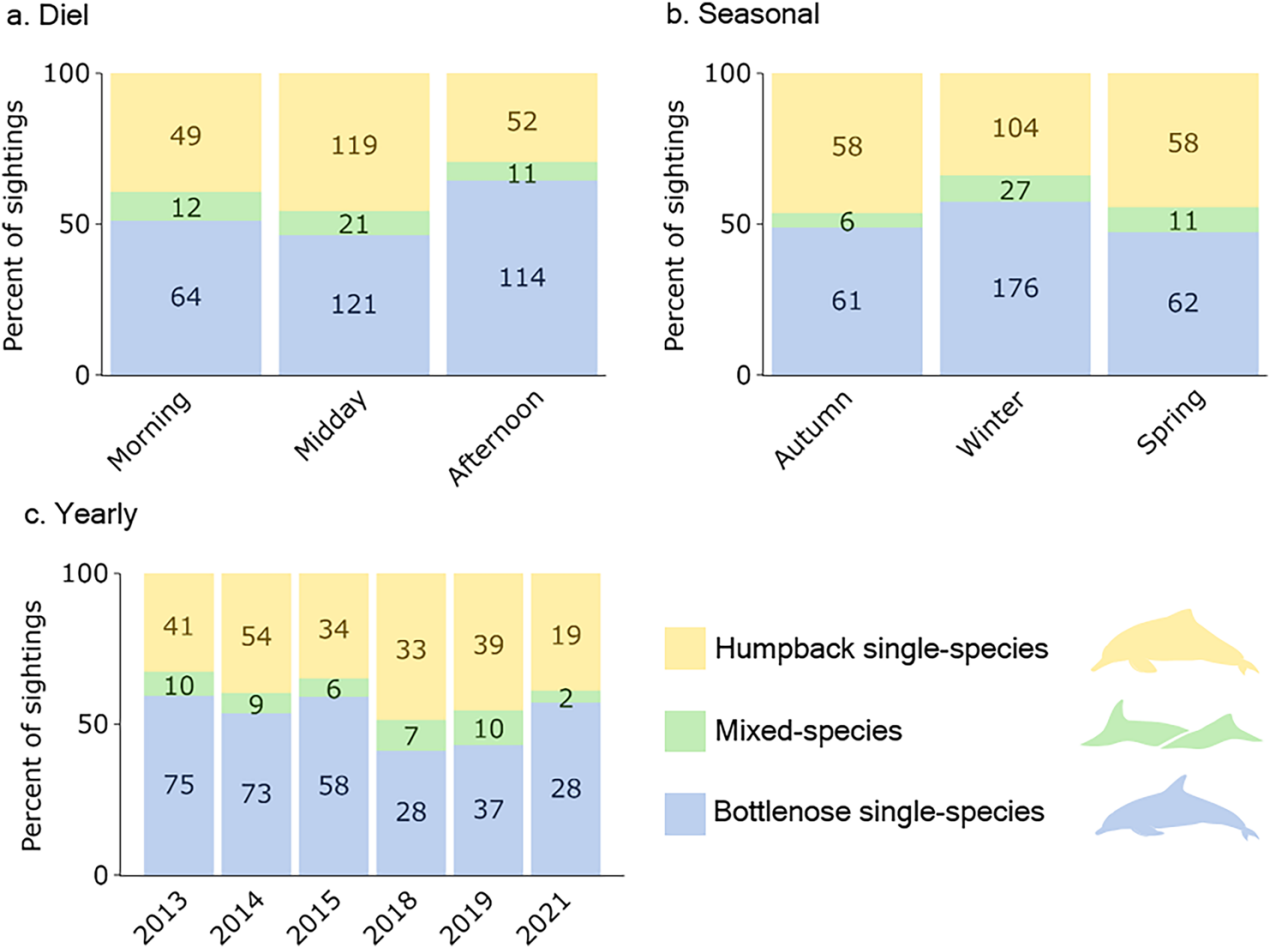
The percentage of on-effort sightings that were of only Australian humpback dolphins (*Sousa sahulensis*), only Indo-Pacific bottlenose dolphins (*Tursiops aduncus*), and mixed-species sightings containing both species across three temporal scales—(**a**) diel: morning (0600 to 1000 h), midday (1000 to 1400 h), and afternoon (1400 to 1900 h); (**b**) seasonal: autumn (March—May), winter (June—August), and spring (September—November); and (**c**) yearly. Numbers in the bars are the total number of sightings for each sighting category in that temporal period.

Single- and mixed-species sightings were observed throughout diel, seasonal, and yearly temporal scales (Fig. 5). We found some evidence for diel temporal partitioning between humpback and bottlenose dolphins (${\chi }_{ 2}^{2}=11.53, p<0.001$), with bottlenose dolphins sighted more often than humpback dolphins during the afternoon. There was, however, no significant differences in the number of humpback and bottlenose dolphins sighted across seasons and years (seasons: ${\chi }_{ 2}^{2}=5.31, p=0.07$; years: ${\chi }_{ 5}^{2}=8.68, p=0.12$). Finally, no temporal variation in the proportion of single- and mixed-species sightings was detected for either species at a diel (humpback: $p=0.69$; bottlenose: $p=0.22$; Fisher's exact test), seasonal (humpback: $p=0.15$; bottlenose: $p=0.52$; Fisher's exact test), or yearly scale (humpback: $p=0.88$; bottlenose: $p=0.28$; Fisher's exact test).

## Discussion

Numerous delphinid species occur in sympatry including many that appear to form mixed-species groups^6–8,37^. Few studies have, however, quantitatively assessed the mechanisms that allow sympatric dolphins to coexist (reviewed in^37^) or determined whether apparent mixed-species groups are indeed the result of attraction between heterospecific individuals and not simply chance encounters or aggregations around resources^6–8^ (but see^38–40^). Sympatric humpback and bottlenose dolphins that live around the North West Cape, Western Australia, display some level of habitat partitioning with regards to water depth and distance to shore. Furthermore, some diel patterns in habitat preferences were also identified. Despite this spatial and temporal partitioning in their habitat use, humpback and bottlenose dolphins displayed a high and positive association after accounting for their shared responses to environmental variables, suggesting that their co-occurrence is not due to chance. We believe that this finding, combined with observations of the species behaving in such a way that they met our operational definition of a group, validates the assumption that they actively form mixed-species groups^6^.

Before considering the implications of these findings, however, it is necessary to consider some potential limitations of the data collection and analyses. Given that dolphins spend most of their time underwater and are highly mobile, false negatives (i.e., not detecting a species when it is there) may have occurred, possibly affecting the observed rates of occurrence. Additionally, mixed-species sightings, which were, on average, larger in size (number of individuals) than single-species sightings of either species and regularly engaged in socialising behaviour^69^, may have been easier to detect, possibly inflating the observed rate of co-occurrence^22^. Nevertheless, these potential sources of bias were minimised by having the data recorded by trained observers under optimal survey conditions (i.e., Beaufort scale ≤ 3, no rain or fog) following a predetermined protocol implemented repeatedly across numerous surveys.

### Habitat partitioning

Humpback and bottlenose dolphins are ecologically similar — both occur in inshore waters and forage on coastal prey^70,71^. To be able to coexist, they presumably partition available resources and habitats, particularly if resources are limiting^2,34,36,37^. Both species were observed in the study area throughout the day and during all three seasons and all six years surveyed, with limited evidence for only diel partitioning in habitat use. This suggests that temporal partitioning of habitat use between humpback and bottlenose dolphins around the North West Cape has a minor role in allowing coexistence. It should be noted, however, that our observations and subsequent analyses of temporal partitioning were limited to daylight hours and seven months of the year (April to October). Moreover, these analyses only considered temporal partitioning of habitat use and did not examine, for example, temporal differences in behaviour, which can also lead to niche partitioning^72^. To gain a comprehensive understanding of temporal niche partitioning, future studies should endeavour to overcome these shortcomings.

We detected stronger patterns of spatial partitioning, however, with humpback dolphins found in shallower water and closer to shore than bottlenose dolphins. This concurs with previous studies of niche partitioning between these species^44,46^. Elsewhere, it has been shown that, although habitat partitioning may occur, niche partitioning is mediated by differences in trophic niches^35,36,73^. Around the North West Cape, the trophic interactions of humpback and bottlenose dolphins are poorly understood, however, opportunistic observations of both species foraging suggest that the species may target different prey. For example, as at other locations^74^, bottlenose dolphins were observed foraging at the surface on epipelagic fish (e.g., *Hemiramphus* sp.), a behaviour that humpback dolphins, which seem to forage mostly on demersal resources, were not observed to perform (JS and GJP personal observations). Further analysis of dietary differences, via stable isotope or stomach content analyses, are required, however, to thoroughly assess potential differences in prey species.

In summary, temporal and habitat partitioning, perhaps combined with the use of different resources, may allow humpback and bottlenose dolphins to coexist around the North West Cape. Yet, humpback and bottlenose dolphins were regularly observed in close spatiotemporal proximity and exhibited a high, positive correlation in occurrence. Similarly, in numerous locations, dolphin species that exhibit niche partitioning have also been observed in apparent mixed-species groups^34,44,73,75,76^.

### Chance encounters?

By analysing presence-absence data with a JSDM, we quantitatively confirmed that, after accounting for shared environmental responses, the co-occurrence of humpback and bottlenose dolphins around the North West Cape is not the result of chance. In doing so, we demonstrate that JSDMs provide an effective means to quantitatively determine whether apparent mixed-species groups are simply chance encounters of sympatric species or not. This is considered a key step in the study of mixed-species groups^6,7,14,15^, but is rarely conducted in studies of delphinid mixed-species groups^6–8^. Although our focus is on the dolphins of the North West Cape and, more broadly, on delphinid mixed-species groups, this approach could, in theory, be applied to species of any taxa that are believed to form mixed-species groups, provided that appropriate presence-absence data are available. Such data are typically readily obtainable, even for wide-ranging marine species, making the use of JSDMs a more feasible approach than previously used techniques, such as ideal gas models, which require detailed data on group dynamics^8,14,20^, and analyses of interspecific social networks, which require individual identification data^38,39^.

Moreover, JSDMs can incorporate environmental factors into analyses of species co-occurrence rates^27–29^, the importance of which was highlighted by our results. Humpback and bottlenose dolphins displayed different, yet overlapping, responses to water depth and distance to shore, indicating that shared habitat preferences may be partially responsible for their co-occurrence. These responses to environmental factors would not have been detected with previously used methods to assess species co-occurrence, such as null models or ideal gas models, that do not incorporate environmental factors^25,27^. Yet, by using a JSDM we identified and accounted for the influence of key environmental factors, revealing a highly positive residual correlation in the occurrence of humpback and bottlenose dolphins.

### Potential drivers for species associations

Residual correlation in the co-occurrence of two species does not equate to evidence for ecological interactions between them^24,25,29^. Instead, interspecific interactions are one possible explanation for observed non-random patterns of co-occurrence, alongside missing environmental or anthropogenic variables and biotic interactions with other species, such as predators and prey^24,25,28,29^.

It is possible that unmeasured environmental factors are responsible for some of the observed residual correlation. However, previous research on the study species has shown that a range of environmental (e.g., benthic habitat type, slope, seabed complexity, sea surface temperature, distance to reef passage, and distance to reef) and anthropogenic (e.g., distance to sanctuary zones and distance to boat ramp) variables have little to no effect on their distribution^45,47^. Therefore, the effect of these covariates on the co-occurrence patterns of humpback and bottlenose dolphins around the North West Cape would presumably be minimal.

The co-occurrence of humpback and bottlenose dolphins could also be explained by biotic interactions. These biotic interactions could take the form of independent interactions between both species and another^29^, for example, mutual avoidance of large sharks could lead to shared use of safer habitats while shared attraction to food pulses could lead to aggregations around schools of fishes^77^. Alternatively, there could be a direct biotic interaction between the two species, such as an attraction between them stemming from evolutionary benefits that they gain by co-occurring^6,7^. Disentangling direct interactions between humpback and bottlenose dolphins, from interactions between these species and their predators or their prey is difficult, particularly if both types of interaction influence patterns of co-occurrence^8,29^. For example, both species may respond similarly to a food pulse because they are independently attracted to the same prey, because they gain some foraging benefit from the other dolphin species, or a combination of both^77,78^. Including the distribution and abundance of predators and prey in JSDMs would help to identify the role that predator–prey dynamics play in determining species distributions and co-occurrence patterns^33^. Where data on predators and prey are unavailable, environmental factors can represent proxies for underlying ecological and spatiotemporal processes such as the distribution, availability, and movement of predators and prey. Yet, the included environmental factors could not fully explain the co-occurrence of humpback and bottlenose dolphins. Moreover, if co-occurrence resulted from shared use of safe habitats, then the species should co-occur primarily within those habitats (e.g., deep areas with sandy substrate^79^). Yet, mixed-species sightings were not limited to any single habitat, instead, they were distributed throughout the study area, from very shallow inshore waters to deeper waters offshore^69^. If co-occurrence resulted from shared attraction to food resources then the species would, presumably, co-occur at times and locations where prey is concentrated (e.g., around schools of prey fish). For example, in the Azores, feeding aggregations of dolphins and tunas occur primarily at dawn and dusk around concentrations of prey fish^77^. This does not appear to be the case around the North West Cape as the species rarely foraged when together and mixed-species sightings occurred throughout space and time^69^. Thus, interactions with predators and prey do not seem to fully explain the co-occurrence patterns of humpback and bottlenose dolphins around the North West Cape.

A remaining possibility is that the positive association in humpback and bottlenose dolphin occurrence is, at least partly, the result of attraction between the two species. This finding concurs with behavioural observations of humpback and bottlenose dolphins apparently engaging in diverse, direct behavioural interactions^69^. Detailed analyses of behavioural data (e.g., behavioural budgets or evaluation of the nature and directionality of behavioural interactions) using a larger sample of sightings will provide further confirmation and indications as to what drives the attraction between the species. Attraction between humpback and bottlenose dolphins, combined with observations of them behaving in such a way that they met the operational definition of group, suggests that they actively form mixed-species groups^6^. Three main functional explanations have been proposed as to why mammals, including dolphins, form mixed-species groups: the antipredator, foraging, and social advantage hypotheses^6–8^. Interestingly, as the temporal analysis did not indicate any diel or seasonal patterns in the occurrence of mixed-species groups, any drivers of their formation are presumably active throughout the day and across seasons. Evidently, further analyses of the characteristics of mixed-species groups (e.g., group size, relative numbers of each species, and sex and age composition) and the behaviours exhibited by the participating individuals are required to determine more precisely what drives sympatric humpback and bottlenose dolphins around the North West Cape to form mixed-species groups. Ultimately, future research should focus on assessing the evolutionary benefits that these species may gain by grouping with heterospecifics.

## Supplementary Information

Supplementary Information.

## Data Availability

The data for this study is stored at Flinders University and can be made available by the authors to any qualified researcher upon reasonable request.
