# Supplementary material for: Habitat partitioning, co-occurrence patterns, and mixed-species group formation in sympatric delphinids
Source: Sci Rep. 2023 Mar 3;13:3599. doi: 10.1038/s41598-023-30694-w (PMC9984456; doi:10.1038/s41598-023-30694-w)
Supplement: Supplementary file 1 — Supplementary Information. [file 41598_2023_30694_MOESM1_ESM.pdf]

Scientific Reports

Supplementary Information: Habitat partitioning, co-occurrence patterns, and mixed-species group formation in sympatric delphinids

Jonathan Syme[1]*, Jeremy J. Kiszka[2], Guido J. Parra[1]

[1]Cetacean Ecology, Behaviour and Evolution Lab, College of Science and Engineering, Flinders University, Adelaide, SA, Australia

[2]Institute of Environment, Department of Biological Sciences, Florida International University, North Miami, FL, United States

*Corresponding author: jonathan.syme@flinders.edu.au

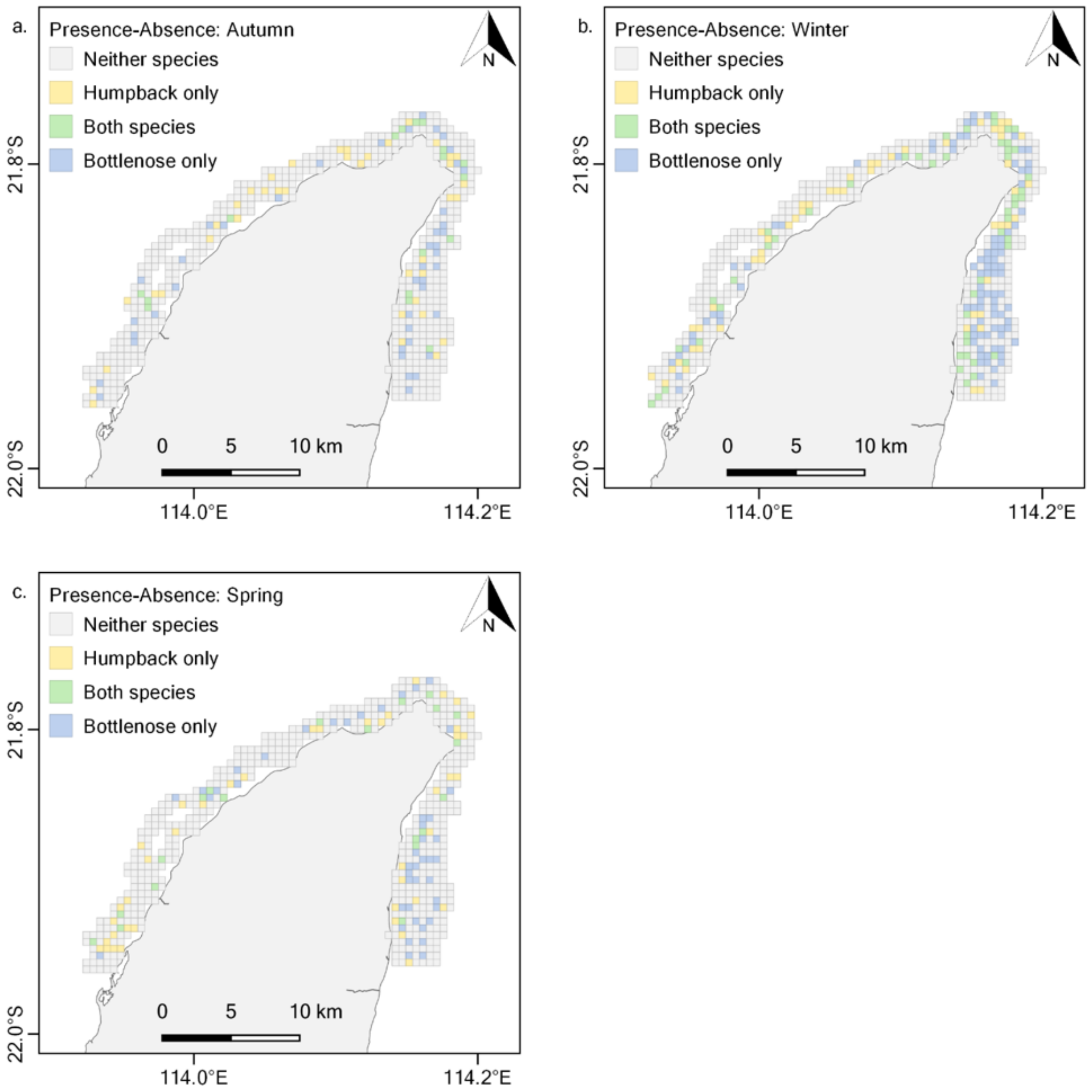

Supplementary Figure S1 The presence-absence values of Australian humpback (*Sousa sahulensis*) and Indo-Pacific bottlenose dolphins (*Tursiops aduncus*) in 540 grids of 500 x 500 m around the North West Cape, Western Australia, across three austral seasons – (a) autumn, (b) winter, and (c) spring – which were used as response variables in a joint species distribution model of their occurrence.

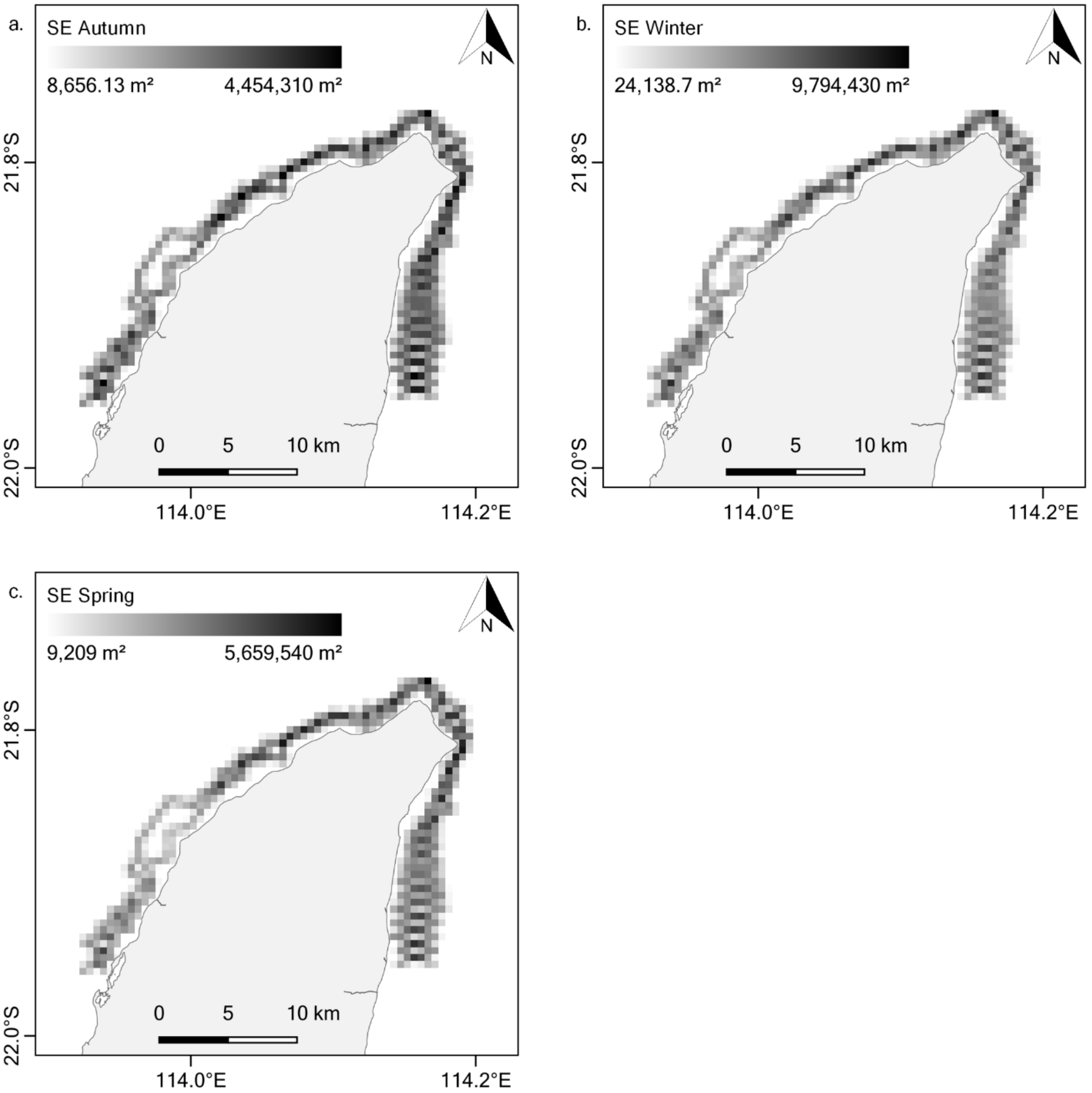

Supplementary Figure S2 Cumulative survey effort (SE) over six years of surveys (2013-2015, 2018-2019, and 2021) across three austral seasons – (a) autumn, (b) winter, and (c) spring – in 540 grids of 500 x 500 m around the North West Cape, Western Australia, which was used as a predictor variable in a joint species distribution model of the occurrence of Australian humpback (*Sousa sahulensis*) and Indo-Pacific bottlenose dolphins (*Tursiops aduncus*). See Table 1 for details on how the values were calculated.

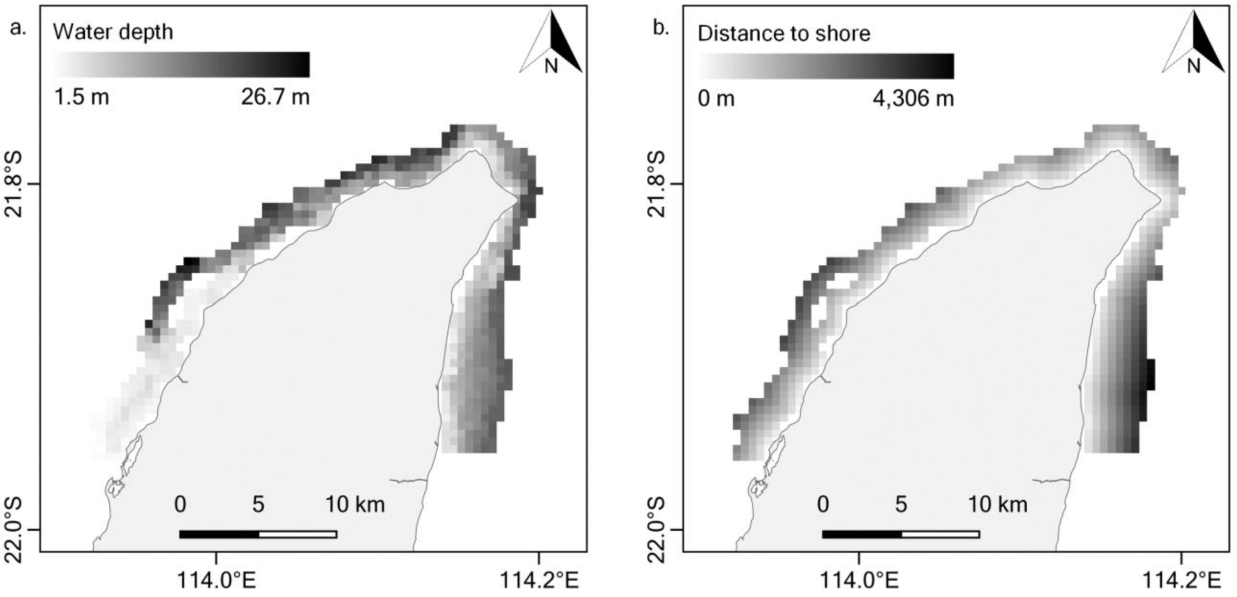

Supplementary Figure S3 The environmental predictor variables – (a) water depth and (b) distance to shore – in 540 grids of 500 x 500 m around the North West Cape, Western Australia, which were included in a joint species distribution model of the occurrence of Australian humpback (*Sousa sahulensis*) and Indo-Pacific bottlenose dolphins (*Tursiops aduncus*). See Table 1 for details on how the values were calculated.

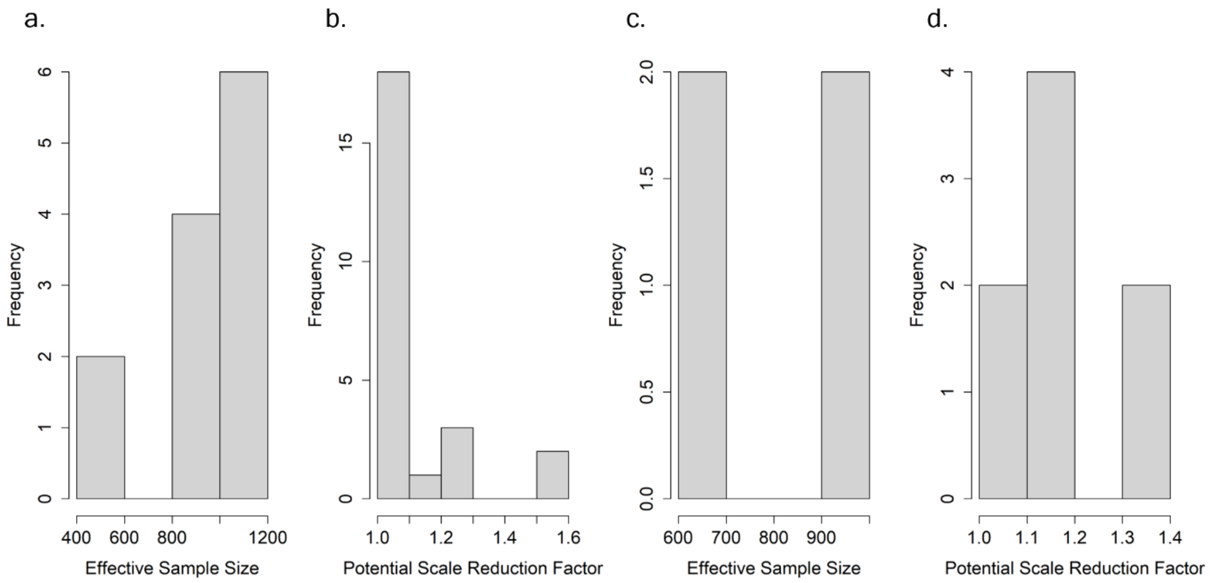

Supplementary Figure S4 Diagnostic histograms for Markov chain Monte Carlo (MCMC) convergence for the joint species distribution model of Australian humpback (*Sousa sahulensis*) and Indo-Pacific bottlenose dolphin (*Tursiops aduncus*) occurrence around the North West Cape, Western Australia. The histograms show the effective sample sizes (a. and c.) and the potential scale reduction factors (b. and d.) for the beta parameters (i.e., the species responses to environmental variables; a. and b.) and the omega parameters (i.e., the species associations at the site level; c. and d.). The theoretical optimum for effective sample sizes is 1000 while potential scale reduction factors below 1.1 indicate a high level of model convergence.
